# On the New Vegetable Alkalies, Veratria, Delphinia, & Aconitine in Neuralgia, and Toothache

**Published:** 1840-06

**Authors:** Samuel A. Cartwright

**Affiliations:** Natchez; Louisville


### WESTERN JOURNAL.

No. 6.

LOUISVILLE, JUNE 30, 1840.

### VERATRINE, DELPHINE AND ACONITINE, *in Neuralgia, Rheumatism,* and *other diseases.* By SAMUEL A. CARTWRIGHT, M. D., of Natchez.

DR. CARTWRIGHT, during his late visit to this city, related to us some striking cases of cure by the external application of these new and most active remedies. At our request, he furnished us with the following communication, which we hope will lead some of our readers to make a trial of the articles, and report to us the results of their experience. Dr. Cartwright has seen acute rheumatism, neuralgia, and tooth-ache effectually and promptly relieved by friction with the ointments; and although he does not profess to have used them extensively in his own practice, his experience strengthens the good opinion he formed of them while witnessing their action in the hospitals of London and Paris. Y.

DEAR SIR:—It is near bed-time, and I leave the city in the morning in quest of health. I have not time to write an essay, but as you took so deep an interest in the cases which I mentioned to you that I had seen treated successfully with certain alkaloids, I herewith leave with you a few extracts from my note-book, compiled from clinical observations made in Europe. As I have nothing original to offer in regard to the use of the new medicines, I think it better to copy my European notes than to enter into any details of my own experience: that experience, as far as it goes, is favorable.

I will, therefore, copy the various formulas in which I have seen these new medicines, in the various hospitals of London and Paris: secondly, enumerate the principal diseases in which they have been used with more or less success; and thirdly, the manner of using them. Although I write in great haste, you may rely on the correctness of the formulas, as I had plenty of leisure while abroad, and was very particular, deliberate and careful in taking my notes.

(1.) *Veratria Ointment.* (Veratrum Sabadilla.)

℞ Veratria ʒss.

Ol. Oliv. ʒj.

Adip. ppt. ℥j. M. fiat ung

*Veratria Embrocation.*

℞. Veratria ʒj.
Sp. rect. ʒj. M.

*Veratria Pills.*

℞. Veratria gr. j.
Extract Hyosciam.
Pulv. glycyrrhiz. aa grs. xij. m. ft. Pil. Xij. Dose, one pill every three hours.

*Delphinia Ointment.* (Delphinium Staphysagria.)

℞. Delphinia ʒss.

Ol. oliv. ʒj.

Adip. ppt. ℥j. M. ft. ung.

*Delphinia Embrocation.*

℞. Delphinia ℈j.

Sp. rect. ℥ij. M. ft. embroc.

*Delphinia Pills.*

As the Veratria pills

*Aconitine Ointment.* [Aconitum Napellus.]

℞· Aconitina grs. xvj.

Ol. oliv. ʒss.

Adip. ppt. ℥j. M.

*Aconitine Embrocation.*

℞. Aconitina grs. viij.
Sp. rect. ℥ij. M.

*Aconitine Pills.*

After the same formula as the veratria and delphinia pills,

The *tincture of aconite* is made by macerating one pound of coarsely powdered aconite root in two pounds of alcohol, seven days, and filtering.

The *delphinia* is made by evaporating to a thin extract a saturated tincture of the seed of the stavesacre, (Delphinium Staphysagria,) and treating it with water acidulated with sulphuric acid, filtering the solution, and precipitating it by ammonia—freeing the precipitate from its water by taking it up by alcohol, and again reducing it to an extract. Then dissolve the extract by acidulated water, and add nitric acid while a precipitate falls. The liquid freed from this precipitate, ammonia is added, and the powder thrown down and dried. It is white.

The extract of *aconite* is made by evaporating the tincture to an extract. The *aconite* pills are made after the same formula as the *aconitine* pills, using twice the quantity of the alcoholic aconite extract in lieu of the aconitine. Twelve aconitine pills contain one grain of aconitine; and twelve aconite pills contain two grains of the extract of aconite. The dose of the former is one twelfth of a grain and of the latter, one-sixth of a grain.

The extract of sabadilla is made by evaporating the tincture of the seeds of the veratrum sabadilla, to the consistence of an extract. The sabadilla pills are made after the formula of the veratria pills, using, however, two grains of the alcoholic extract of sabadilla, in place of one grain of veratria.

The *tincture of stavesacre* is made after the same manner as the tincture of sabadilla, using the powdered seed of the stavesacre, for those of the sabadilla.

The *ioduretted veratrine ointment* is made by substituting half a drachm of the hydriodate of potash for the olive oil, in the preparation of the veratria ointment.

2. The diseases in which some one or more of the foregoing preparations have been found more or less useful, are tic douloureux rheumatism, acute and chronic, croup, angina pectoris, gout, dropsy, spasms of the stomach, tooth-ache, scrofulous swellings, capsular cataracts, iritis, internal opthalmia, amaurosis, and hypertrophy of the membranes in general, and a variety of indurations, thickenings and swellings.

3. *Manner of using the remedies.*—The hand is to be kept moist with any one of the ointments, and rubbed briskly, and with some little force, over the affected part for ten or fifteen minutes, or more. There is no fear, says Mr. Turnbull, of London, of using the friction too long. He assured us that when used beyond a certain point, the electro-stimulation became unsupportable. This stimulation, he contended, must be fully induced before the sanative effect of the remedy can be expected. What he calls *electro-stimulation*, is a sense of pricking and numbness in the parts subjected to the friction. The ointments do not chafe or irritate the skin. They are applied generally two or three times a day, but in violent cases they are used oftener—say, every three hours, until the electro-stimulation is fully induced, and the disease relieved; then to be gradually discontinued.

The tinctures and embrocations are used pretty much after the same manner with the ointments. The internal use of the medicines did not fall under my observation; but when the electro-stimulation cannot be sufficiently induced by their external use, the pills are advised both by the London and Paris physicians—one, for example, every three hours.

The several preparations above mentioned, have nearly the same effects. The veratrine, delphine and aconitine ointments, embrocations and tinctures, are alternately used, to induce, in the first place, the electro-stimulation, and, in the second, to keep up that stimulation. Some persons are not affected by one, when they would be by another; and the same preparation after being continued for some time, loses its effect in some cases, and hence recourse should be had to another. In croup, the ointment is rubbed over the throat—in neuralgia, over the affected nerve.

One of the surgeons of St. Bartholomew's hospital, in a case of tic douloureux, made an ointment of aconitine, five grains, rubbed with five drachms of cerate. He applied a very small quantity with his finger over the painful nerve, once or twice a day, for six days. It produced numbness over the jaw for twelve or eighteen hours. The aconitine was prepared by Mr. Mordon, of Southampton-row, London, who is said to make the purest article.

In affections of the eyes, the ointment is rubbed over the forehead. The ointments are applied with the hand: a sponge is used in applying the tinctures and embrocations. The medicines are all very costly, and are apt to be adulterated, or improperly prepared. When genuine, they nearly always promptly produce a degree of numbness, and a pricking sensation on the surface to which they are applied. Their application to blistered surfaces, and to the mucous membranes had been ventured on, but great caution is necessary in this mode, as the experience with them thus applied, is not sufficient to enable us to form rules.

When I was in London, Dr. Turnbull was preparing a work on the above remedies. S. A. C.

*Louisville,* June 16, 1840.

